# The CRAFITY score emerges as a paramount prognostic indicator in hepatocellular carcinoma patients received Lenvatinib and Pembrolizumab

**DOI:** 10.3389/fimmu.2024.1474456

**Published:** 2024-11-01

**Authors:** Weijie Wu, Zhenyun Yang, Hao Zou, Teng Long, Zhongguo Zhou, Yaojun Zhang, Minshan Chen, Dandan Hu

**Affiliations:** ^1^ Department of Liver Surgery, Sun Yat-sen University Cancer Center, Guangzhou, Guangdong, China; ^2^ Collaborative Innovation Center for Cancer Medicine, State Key Laboratory of Oncology in South China, Sun Yat-Sen University Cancer Center, Guangzhou, Guangdong, China; ^3^ Guangdong Provincial Clinical Research Center for Cancer, Sun Yat-Sen University Cancer Center, Guangzhou, Guangdong, China

**Keywords:** hepatocellular carcinoma, lenvatinib, pembrolizumab, C-reactive protein and A-fetoprotein in immunotherapy score, overall survival

## Abstract

**Background:**

Levels of C-reactive protein (CRP) and alpha-fetoprotein (AFP) in immunotherapy (CRAFITY) scores are associated with the prognosis of patients with hepatocellular carcinoma (HCC). This study aimed to explore the efficacy of lenvatinib and pembrolizumab (Len-P) based on the CRAFITY score.

**Methods:**

In this study, 228 patients with HCC who received Len-P in Sun Yat-sen University Cancer Center were included. CRAFITY 0 score was defined as AFP level below 100 ng/ml, CRP level below 1 mg/dl, CRAFITY 1 score was defined as AFP level at least 100 ng/ml or CRP level at least 1 mg/dl. CRAFITY 2 scores were defined as AFP levels exceeding 100 ng/ml and CRP levels exceeding 100 ng/ml. The primary outcome was overall survival (OS). The second outcome was tumor response rate.

**Results:**

The survival time of CRAFITY 0 is significantly longer than that of CRAFITY 1 and CRAFITY 2 (p =.044). Univariate analysis showed that largest tumor size (HR = 2.149; 95% CI 1.129 - 4.091; p =.02), lymph node metastasis (HR = 2.012; 95% CI 1.132- 3.579; p = .017), and CRAFITY (HR = 0.372; 95% CI 0.168-0.824; p = .015) were important risk determinants of OS in all patients. The results of multivariate analysis show that CRAFITY score is an independent risk factors for OS (HR = 0.719; 95% CI 0.377-1.374; p =.048). The ORR of CRAFITY 0, 1 and 2 scores were 36.4%, 32% and 27.4%, respectively (*p* = .556). The ORR of intrahepatic lesions by CRAFITY 0, 1 and 2 were 37.9%, 35%, 30.6% (*p*= .688).

**Conclusion:**

CRAFITY score is a good predictor of prognosis in HCC patients receiving Len-P.

## Introduction

Hepatocellular carcinoma (HCC) is one of the most common gastrointestinal malignancies in the world (1). It is estimated that between 2020 and 2040, the annual new cases of liver disease will increase by 55%, and 1.4 million people may be diagnosed with liver cancer in 2040, and 1.3 million people may die from liver cancer in 2040 (2). Regrettably, due to the insidious onset of HCC, most of patients are diagnosed initially at an intermediate or advanced stage, thereby constraining treatment options primarily to non-surgical interventions (3). Systemic therapy is the first choice for patients with advanced HCC. The first-line treatment strategy for advanced HCC has shifted from anti-angiogenic tyrosine kinase inhibitor (TKI) monotherapy to immunotherapy-based combination therapy (4).

Lenvatinib, an oral small-molecule multi-receptor TKI, has been approved as a first-line treatment for patients with unresectable liver cancer in the United States, the European Union, Japan and China. In the Phase 3 REFLECT study, the overall survival rate of lenvatinib was no less than that of sorafenib (5). Based on the results of the Phase 2 KEYNOTE-224 study, the U.S. Food and Drug Administration approved pembrolizumab (an anti-PD-1 antibody) for use in patients with advanced hepatocellular carcinoma who had previously received Sorafenib (6). In a KEYNOTE-394 study in Asia, pembrolizumab significantly extended overall survival and progression-free survival of HCC patients (7). In a Phase 1b study, the combination of lenvatinib and pembrolizumab demonstrated favorable antitumor activity in first-line therapy with an objective response rate of 36.0% (95% CI 26.6-46.2) (8). In the LEAP-002 study, median overall survival was 21.2 months (95% CI 19.0–23.6) with lenvatinib plus pembrolizumab versus 19.0 months (95% CI 17.2–21.7) with lenvatinib plus placebo (hazard ratio [HR] 0.84; 95% CI 0.71–1.00; stratified log-rank p=0.023) (9).

The CRAFITY score based on serum C-reactive protein (CRP) and alpha-fetoprotein (AFP) levels is an easy-to-apply prognostic score for HCC patients treated with anti-PD-1-based immunotherapy (10). Moreover, CRAFITY score has been shown to be effective in predicting the survival of HCC patients receiving Atezolizumab combined with Bevacizumab (11). However, the prediction of the CRAFITY score’s response to Len-P has not been established. The use of this treatment regimen is expected to increase in the future, so the predictive performance of the CRAFITY score for this treatment is of particular interest.

The objective of this study was to investigate the efficacy of Len-P immunotherapy of HCC patients, and to evaluate the association of CRAFITY score with the prognosis of HCC patients.

## Materials and methods

### Patients

**Figure 1:**
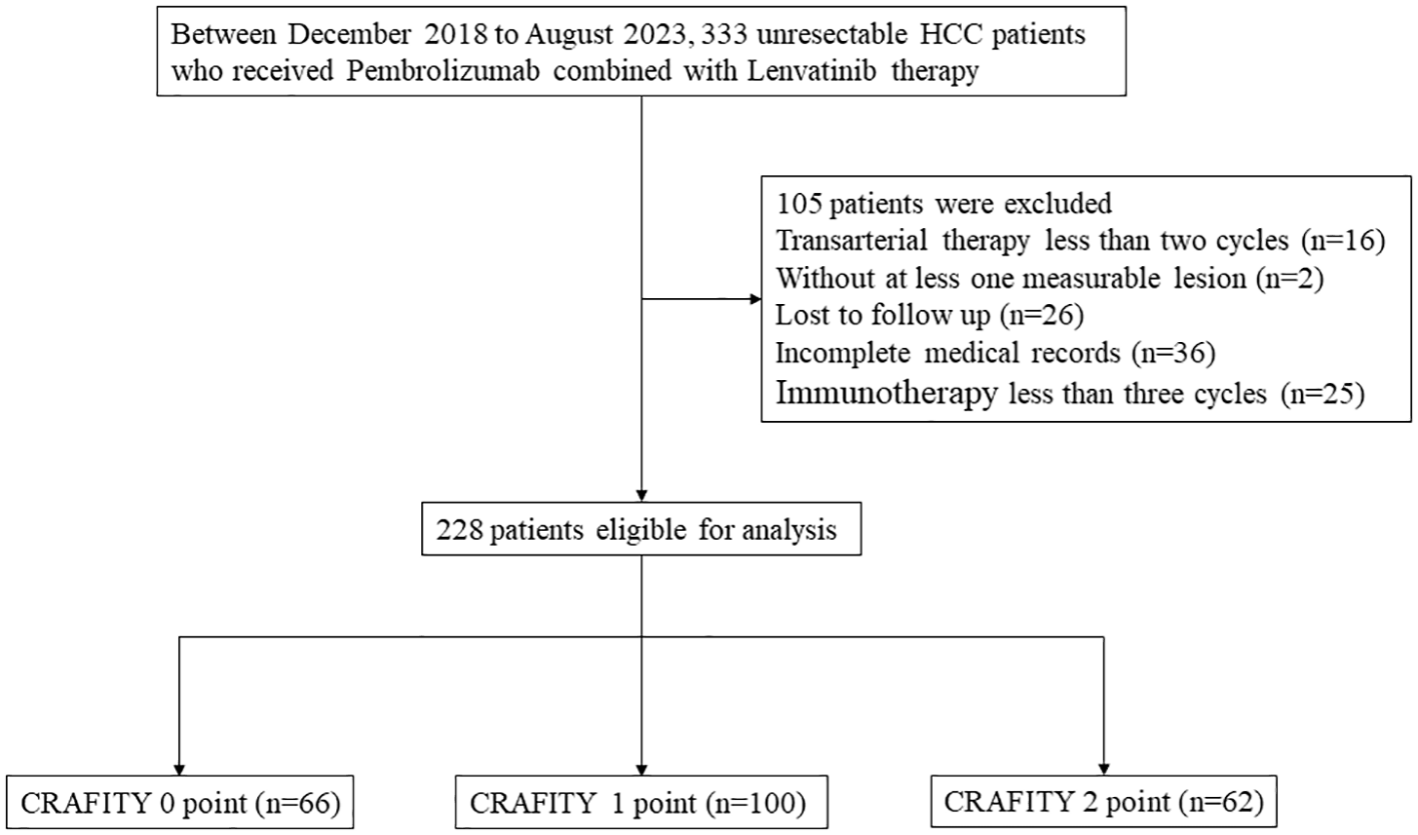
Flow chart of the disposition process of HCC patients. HCC, hepatocellular carcinoma. CRAFITY, C-reactive protein and alpha-fetoprotein in immunotherapy.

The study included 317 patients who received pembrolizumab combined with lenvatinib therapy at Sun Yat-sen University Cancer Center from October 2018 to August 2023. Patients were selected based on the following criteria: (a) imaging or pathological diagnosis of HCC according to the American Association for the Study of Liver Diseases (AASLD) Practice guidelines (12); (b) Patients had received at least 3 cycles of Len-P; (c) Complete AFP and CRP test data are available; (d) There is at least one measurable lesion according to the Modified Response Evaluation Criteria in Solid Tumors (mRECIST) (13). Patients with poor liver function (Child-Pugh C) or other malignancies and with no follow-up data were excluded. Finally, 228 HCC patients were included in the study. The research process is shown in **Figure 1**.

### Treatment procedure

Lenvatinib is administered according to the following regimen: The recommended daily dose of lenvatinib for patients weighing less than 60 kg is 8 mg once daily. For patients weighing 60 kg or more, the recommended daily dose of lenvatinib is 12 mg once daily.

Treatment is continued until the disease progresses or an intolerable toxic effect occurs. Pembrolizumab is administered intravenously every 3 weeks and is started on the same day that transarterial therapy ends.

Transarterial therapy comprised two approaches: hepatic arterial infusion chemotherapy (HAIC) and transhepatic arterial chemotherapy with embolization (TACE). HAIC utilized a combination of 5-fluorouracil and oxaliplatin (FOLFOX). The interval for HAIC was 3 weeks, while for TACE, it was 6 weeks. Efficacy assessments were conducted after 6 weeks.

Within the patients who received Lenvatinib and Pembrolizumab plus transarterial therapy, the interval between transarterial therapy and immunotherapy is within 1 day. As long as there is no focal progression or intolerable toxicity, lenvatinib and pembrolizumab immunotherapy continues. All patients underwent contra-enhanced computed tomography (CT), magnetic resonance imaging (MRI), and peripheral blood tests every 3 months for 5 years.

### Data collection

Basic statistical measures and clinical characteristics of the patients were collected, including age, gender, AFP and CRP within 4 weeks before starting Len-P therapy. The CRAFITY score was composed of serum AFP and CRP levels at baseline (10). CRAFITY 0 was defined as AFP levels below 100 ng/ml and CRP levels below 1 mg/dl. CRAFITY 1 scores are defined as AFP levels exceeding 100 ng/ml or CRP levels exceeding 1 mg/dl. CRAFITY 2 scores are defined as AFP levels of at least 100 ng/ml and CRP levels of at least 1 mg/dl. In addition, we calculated a series of inflammatory prognostic scores composed of systemic inflammatory response factors, which include platelet to lymphocyte ratio (PLR) (14), neutrophil to lymphocyte ratio (NLR) (15), lymphocyte to C-reactive protein ratio (LCR) (16), lymphocyte-to-monocyte ratio (LMR) (17), systemic immunoinflammatory Index (SII) (18), C-reactive protein to albumin ratio (CAR) (19), the Glasgow prognostic Score (GPS) (20), prognostic index (PI) (21), prognostic nutritional index (PNI) (22). All showed strong predictive power in cancer prognosis.

OS is the interval from the start of Len-P therapy to death or the last follow-up. The Response Evaluation Criteria in Solid Tumors (RECIST) 1.1, including complete response (CR), partial response (PR), stable disease (SD), and progressive disease (PD), were employed for the evaluation of tumor response.

### Statistical analysis

Continuous variables with abnormal distribution were expressed as median [interquartile range (IQR)] and analyzed by the Wilcoxon rank sum test. Disaggregated data are presented as numbers (percentages) and evaluated by Pearson Chi-square test or Fisher precision test. Survival differences between patients with different CRAFITY scores were shown by the Kaplan-Meier curve and assessed by a log-rank test. Multivariate Cox proportional hazard regression models were used to determine the predictors of OS. The predictive power of the CRAFITY score and other inflammatory markers was compared using the area under the curve (AUC) of the time-dependent receiver Operating characteristic (ROC) curve. All statistical analyses were performed using SPSS software (version 25.0; IBM Corp) and R (version 4.0.2). A two-tailed *P*-value of less than 0.05 is considered statistically significant.

## Results

### Characteristics of patients

**Table 1** presents the baseline characteristics of 228 HCC patients with a median age of 55.5 years, and most of patients (91.7%) were male. More than half (84.2%) of patients had a history of hepatitis B virus infection. There were 170 (74.6%), 45 (19.7%) and 13 (5.7%) patients with Child-Pugh scores of 5, 6 and ≥7, respectively. More than half (51.3%) of these patients did not have a portal vein cancer thrombus (PVTT). 106 (46.5%) patients had cirrhosis and 75 (32.9%) had extrahepatic metastases. More than half (79.4%) of patients had stage C of Barcelona Clinical Liver Cancer (BCLC). The median maximum diameter of the tumor was 45 mm. Of these, 124 patients (54.4%) received transarterial therapy. 48.7% of patients had baseline AFP levels of at least 100ng/ml, and 46.9% had baseline CRP levels of more than 1mg/dl. Of these, 66 patients were assigned to CRAFITY 0, 100 to CRAFITY 1, and 62 to CRAFITY 2. **Table 1** indicated that CRAFITY score was closely correlated with Child-Pugh score, portal vein cancer thrombus, BCLC stage, tumor diameter, and transarterial therapy (all *p* <.05).

**Table 1:** Baseline clinicopathological characteristics of HCC patients.

Variables	Primary cohort (n=228)	CRAFITY 0 points (n=66)	CRAFITY 1 points (n=100)	CRAFITY 2 points (n=62)	*p* value
Age (years-old)	55.5 [48.75,62]	56.5 [51, 64.5]	57 [48, 62]	53.5 [47, 58]	0.201
Gender					0.102
Male	209 (91.7)	57 (86.4)	92 (92)	60 (96.8)	
Female	19 (8.3)	9 (13.6)	8 (8)	2 (3.2)	
ECOG PS					0.489
0	158 (69.3)	49 (74.2)	69 (69)	40 (64.5)	
1-2	70 (30.7)	17 (25.8)	31 (31)	22 (35.5)	
HBV infection					0.652
Yes	192 (84.2)	56 (84.8)	86 (86)	50 (80.6)	
No	36 (15.8)	10 (15.2)	14 (14)	12 (19.4)	
Child-Pugh score					0.019
5	170 (74.6)	53 (80.3)	79 (79)	38 (61.3)	
6	45 (19.7)	12 (18.2)	17 (17)	16 (25.8)	
≥7	13 (5.7)	1 (1.5)	4 (4)	8 (12.9)	
Portal vein thrombosis					0.001
Vp1	12 (5.3)	3 (4.5)	6 (6)	3 (4.8)	
Vp2	37 (16.2)	8 (12.1)	18 (18)	11 (17.7)	
Vp3	55 (24.1)	8 (12.1)	22 (22)	25 (40.3)	
Vp4	7 (3.1)	0 (0)	6 (6)	1 (1.6)	
No	117 (51.3)	47 (71.3)	48 (48)	22 (35.6)	
Cirhosis					0.073
Yes	106 (46.5)	23 (34.8)	50 (50)	33 (53.2)	
No	122 (53.5)	43 (65.2)	50 (50)	29 (46.8)	
Extra-hepatic metastasis					0.475
Yes	75 (32.9)	19 (28.8)	32 (32)	24 (38.7)	
No	153 (67.1)	47 (71.2)	68 (68)	38 (61.3)	
BCLC stage					<0.001
A	9 (3.9)	6 (9.1)	3 (3)	0 (0)	
B	38 (16.7)	20 (30.3)	12 (12)	6 (9.7)	
C	181 (79.4)	40 (60.6)	85 (85)	56 (90.3)	
Tumor diameter (mm)	45 [9.575, 95]	12.5 [2.425, 51.75]	46.5 [10.525, 95]	92 [18, 129.5]	<0.001
AFP≥100ng/ml	117 (51.3)	0 (0)	55 (55)	62 (100)	<0.001
CRP≥1mg/dl	107 (46.9)	0 (0)	45 (45)	62 (100)	<0.001
Plus transarterial therapy					0.613
No	104 (45.6)	31 (47)	48 (48)	25 (40.3)	
YES	124 (54.4)	35 (53)	52 (52)	37 (59.7)	
Cycles of immunotherapy	5 [3, 9]	6 [4, 9]	5.5 [3, 9]	4 [3, 7.75]	0.693

Values are presented as the median (IQR) or n (%). *P*-value < 0.05 is statistically significant.

ECOG PS, Eastern Cooperative Oncology Group performance status; HBV, hepatitis B virus; BCLC, Barcelona Clinic Liver Cancer; AFP, alphafetoprotein; CRP, C-reactive protein.

### Overall survival

**Figure 2:**
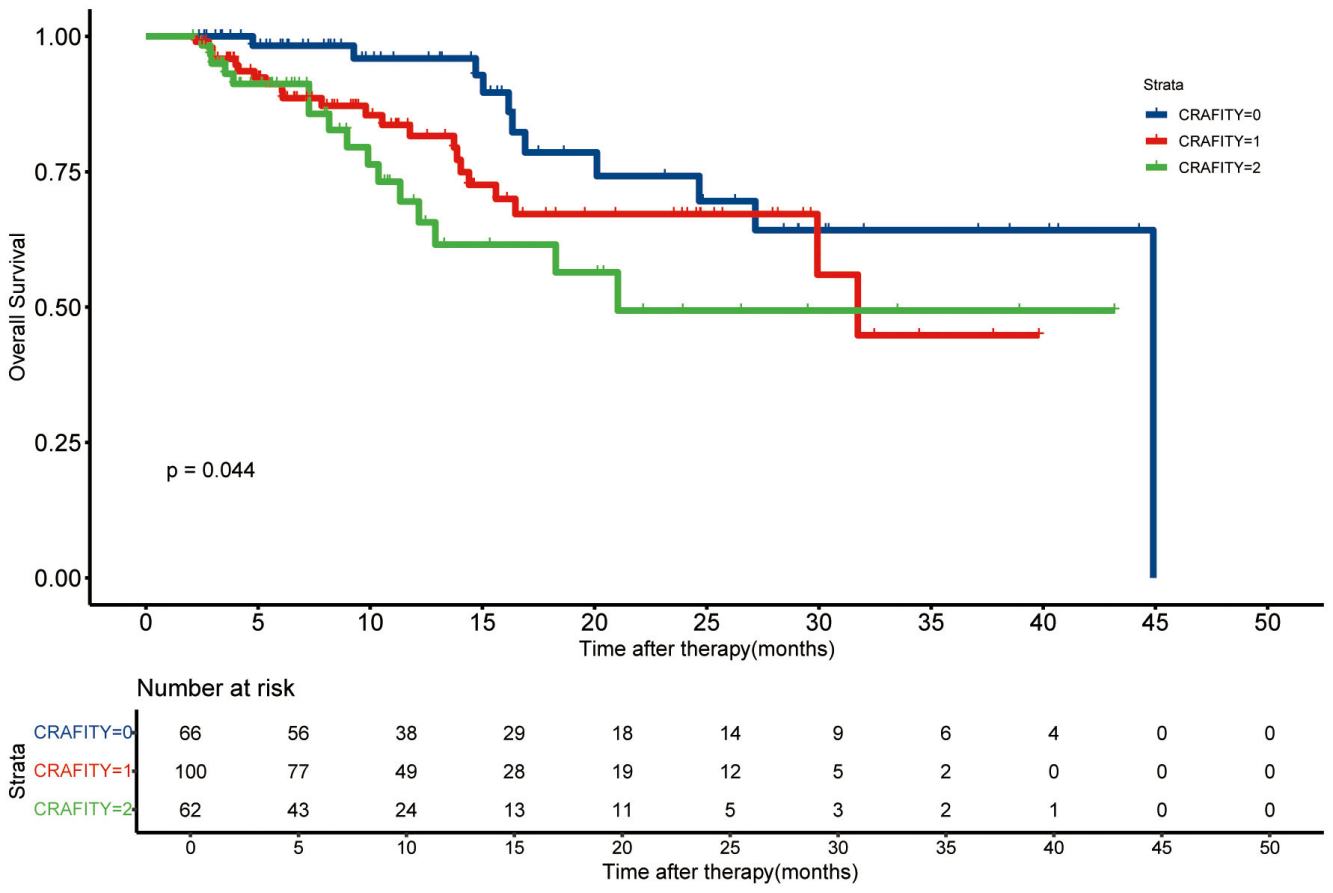
Kaplan-Meier curves of the overall survival based on the CRAFITY score. CRAFITY, C-reactive protein and alpha-fetoprotein in immunotherapy.

The average follow-up time was 15.5 months (95%CI: 13.9 – 17.1 months). We compared the survival time between different CRAFITY rating groups (**Figure 2**). The survival time of CRAFITY 0 is significantly longer than that of CRAFITY 1 and CRAFITY 2 (p =.044). Therefore, CRAFITY score can predict the prognosis of HCC. Other inflammation-based scores were associated with OS in patients who received Len-P therapy (**Supplementary Figure 1**). Low CRP, PLR, NLR, LCR, LMR, SII, CAR, GPS, PI, PNI scores suggested a good prognosis (all P <.05).

### Tumor response on imaging examinations

**Figure 3:**
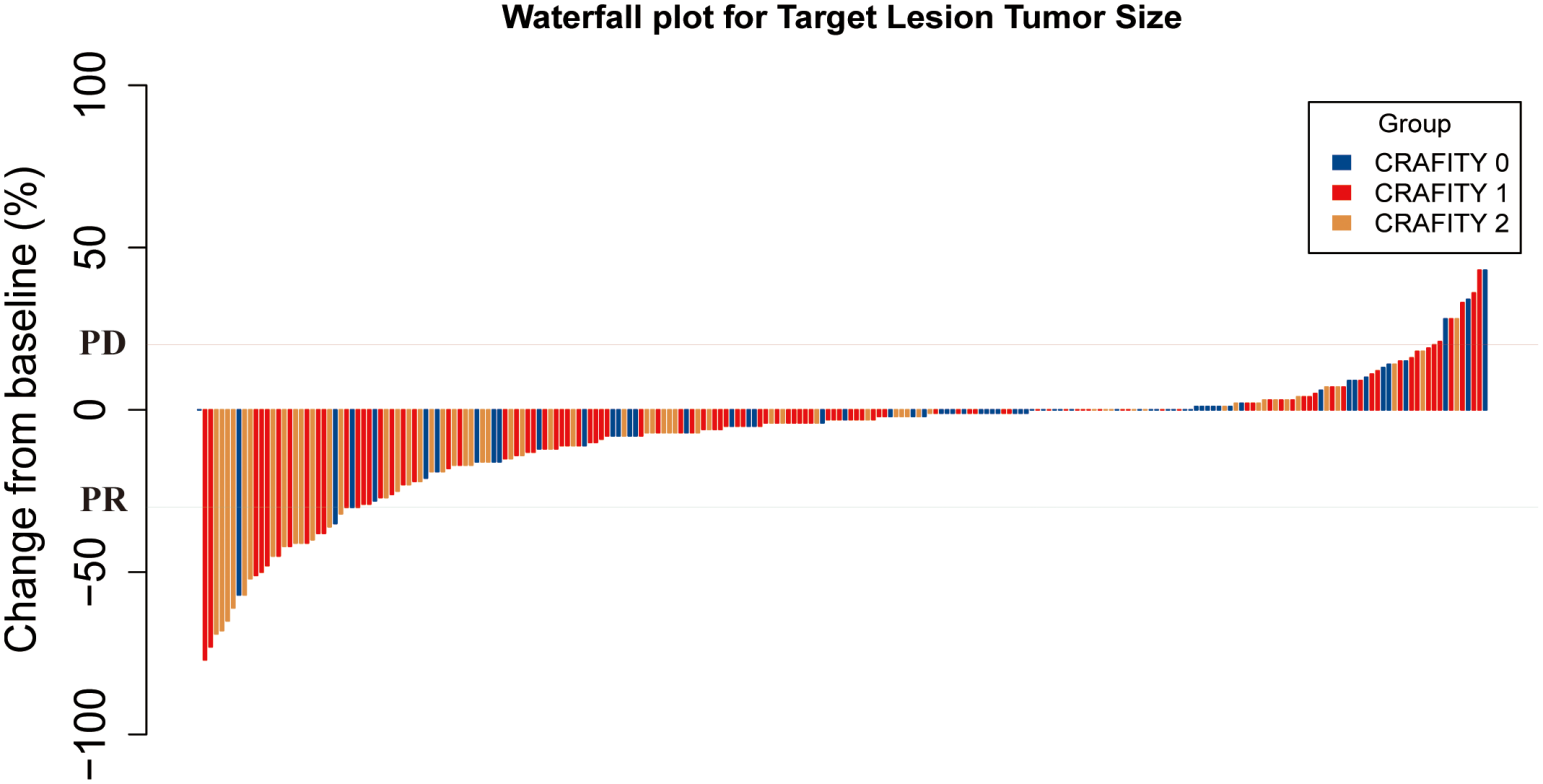
Waterfall plot for tumor size changes of intra-hepatic target lesions. PD, progressive disease; PR, partial response. CRAFITY, C-reactive protein and alpha-fetoprotein in immunotherapy.

**Supplementary Table 1** summarizes the radiological assessment of treatment response. 6, 57, 89 and 76 patients achieved CR, PR, SD and PD, respectively. The ORR and DCR of the entire cohort were 27.6% and 66.7%, respectively. The ORR of CRAFITY 0, 1 and 2 were 36.4%, 32% and 27.4%, respectively (*p* = .556). The DCR of CRAFITY 0, 1 and 2 were 62.1%, 73% and 61.3%, respectively (*p* = .199). The ORR and DCR of intrahepatic response by CRAFITY 0, 1 and 2 were 37.9% vs 35% vs 30.6% (*p*=.688) and 70% vs 77% vs 69.4% (*p*=.453), respectively. Although there was no significant difference in ORR, the CRAFITY 0 group still had a higher ORR than the other two groups. The optimal response for intra-hepatic target lesions by patient according to RECIST1.1 criteria was illustrated in the waterfall plot in **Figure 3**.

### Univariate and multivariate Cox regression analyses in the cohorts

Univariate and Multivariate Cox proportional hazard regression model was used to determine the prognostic factors of OS. Univariate analysis showed that Largest tumor size (HR = 2.149; 95% CI 1.129 - 4.091; *p* =.02), Lymph node metastasis (HR = 2.012; 95% CI 1.132- 3.579; *p* = .017), and CRAFITY (HR = 0.372; 95% CI 0.168-0.824; *p* = .015) were important risk determinants of OS in all patients. As shown in **Table 2**, the results of multivariate analysis show that CRAFITY score is an independent risk factors for OS (HR = 0.719; 95% CI 0.377-1.374; *p* =.048).

**Table 2:** Univariate and multivariate Cox Regression analyses of Risk Factors for Overall Survival.

Variables	Univariate			Multivariate		
	HR	95%CI	*P*-value	HR	95%CI	*P*-value
Age (>/≤50)	1.006	0.532-1.904	0.984			
Gender (female/male)	0.693	0.274-1.753	0.439			
Largest tumor size (>/≤10)	2.149	1.129-4.091	0.02			
Tumor number (>/≤1)	1.734	0.809-3.718	0.157			
Lymph node metastasis (yes/no)	2.012	1.132-3.579	0.017			
Metastasis (yes/no)	1.524	0.853-2.72	0.154			
CRAFITY						
0	Reference			Reference		
1	0.372	0.168-0.824	0.015	0.719	0.377-1.374	0.048
2	0.695	0.364-1.324	0.268	1.644	0.905-2.986	0.318

*P*-value < 0.05 is statistically significant in both univariate and multivariate analyses.

CRAFITY, C-reactive protein and alpha-fetoprotein in immunotherapy.

### Comparison of the predictive power of the CRAFITY score and other inflammation markers

**Figure 4:**
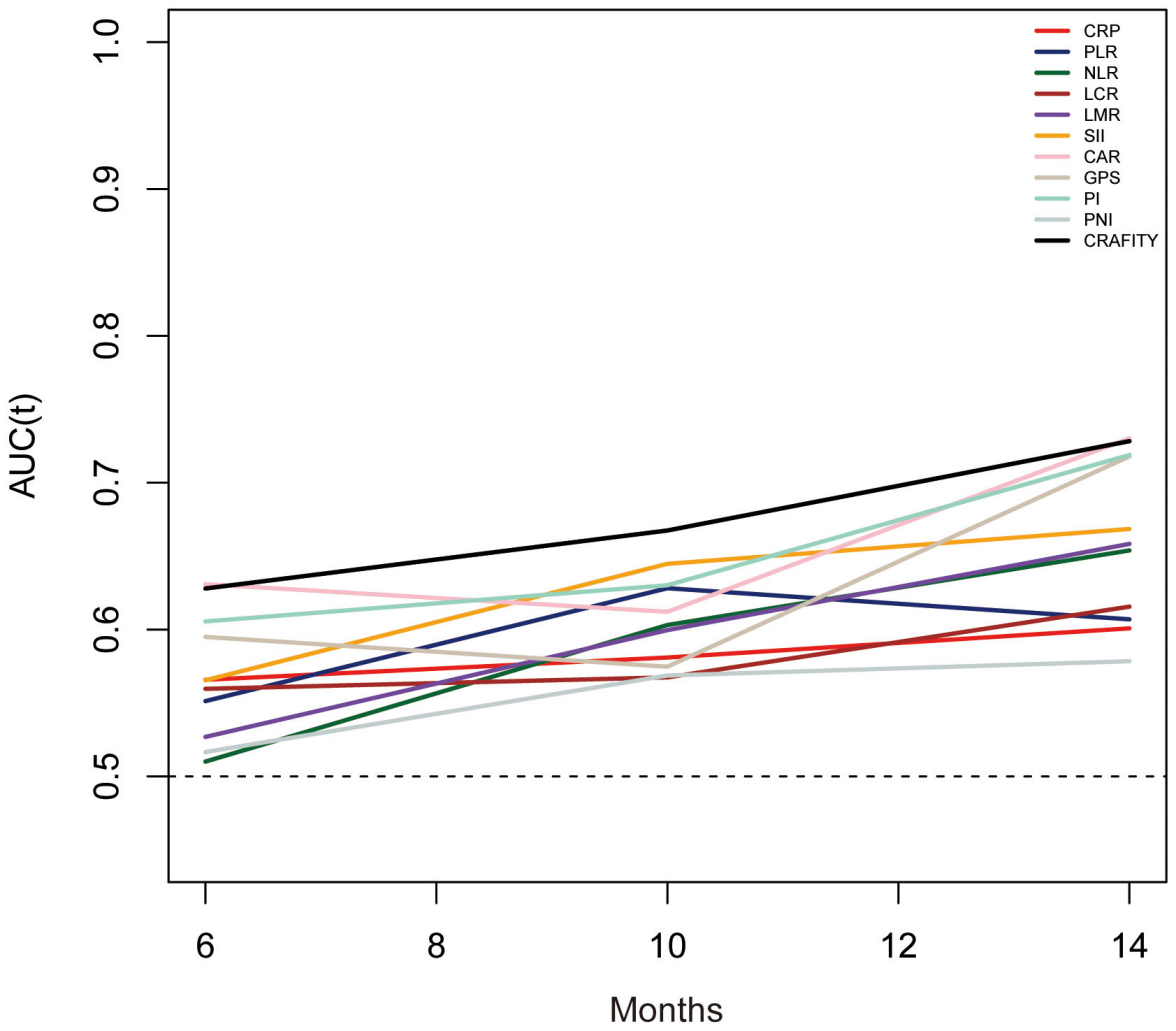
Time-dependent ROC analysis of the CRAFITY score and other infammation markers for overall survival. ROC, receiver operating characteristics, CRAFITY, C-reactive protein and alpha-fetoprotein in immunotherapy.

The predictive power of the CRAFITY score and other inflammatory markers was evaluated using time-dependent ROC curves. The results showed that the CRAFITY score could more accurately predict OS in HCC patients receiving Lenvatinib plus Pembrolizumab immunotherapy than other inflammatory markers (**Figure 4**). The CRAFITY score predicts an AUC of 0.663, 0.668 and 0.689 for OS at 5, 10 and 15 months, respectively (**Supplementary Table 2**).

## Discussion

Combination therapy is the main treatment method for patients with advanced liver cancer, and more and more of them are treated with Len-P. Therefore, it is necessary to use biomarkers to identify those who may benefit from Len-P. Our study found that CRAFITY score can predict the prognosis of patients receiving Len-P, with significant differences in OS between different CRAFITY score groups. Moreover, compared with other inflammatory markers, CRAFITY score can more accurately predict OS in HCC patients treated with Len-P.

Based on baseline AFP and CRP levels, 228 HCC patients treated with Len-P were divided into CRAFITY 0 (n=66), CRAFITY 1 (n=100), and CRAFITY 2 (n=62) groups. The study suggests that higher CRAFITY scores are associated with poorer OS. The results were similar to previous studies.

CRAFITY score is widely used in clinical trials. Studies have shown that CRAFITY score can predict the survival and prognosis of patients receiving PD-L1 immunotherapy (10). The CRAFITY score is an ideal and useful predictor for unresectable HCC patients receiving atezolizumab combined with bevacizumab (23). Yi Yang et al. have also demonstrated that CRAFITY score can predict the prognosis of HCC patients receiving TKI and immunotherapy (24).

It is reasonable to use the CRAFITY score to predict the outcome of HCC patients treated with Len-P. As we all know, one of the hallmarks of cancer is inflammation, which promotes tumor occurrence and progression (25). CRP, as an acute phase protein, can be induced by factors such as interleukin-1 (IL-1), interleukin-6 (IL-6), and tumor necrosis factor-α (TNF-α). It is often used to evaluate the efficacy of tumor treatment, such as predicting PD-L1 expression and immunotherapy effect in non-small cell lung cancer (26, 27). High CRP levels often indicate an immunosuppressive tumor microenvironment, and CRP can induce the infiltration of M2 macrophages and regulatory T cells (28) and inhibit the proliferation and function of CD4^+^ and CD8^+^T cells (29). In addition, AFP, as a biomarker to assist in the diagnosis of HCC, is commonly used to predict HCC recurrence and disease progression (30). The study about serum α-fetoprotein to predict the prognosis of bevacizumab plus immunotherapy in hepatocellular carcinoma have found that the reduction of serum AFP level after anti-PD-1 treatment is associated with a good prognosis for advanced HCC (31). Therefore, it can be considered that AFP may be related to immune escape of HCC, and some studies have shown that AFP may impair the function of macrophages, thereby leading to the decline of antigen presentation and phagocytosis (32). AFP can also cause dendritic cell dysfunction, which affects the activation and differentiation of T cells in HCC (33). Therefore, CRAFITY score can be used to evaluate the immune microenvironment and invasion degree of HCC.

Although CRAFITY 0 had the highest ORR based on intrahepatic response compared to the other two groups, the difference in ORR between CRADITY 0, 1 and 2 was not significant, most likely due to the insufficient number of cases. The number of enrolled cases needs to be increased in subsequent studies to further supplement ORR confidence.

Due to incomplete medical records, we were unable to quantify treatment-related adverse events (AEs) in our study. However, referring to previous literature on the relationship between the CRAFITY score and liver cancer, higher CRAFITY scores have been associated with an increased likelihood of adverse events. For instance, Renguo Guan et al. found that patients with a CRAFITY 2 score were more prone to fever compared to those with other scores, and that higher CRAFITY scores correlated with a greater incidence of Grade 3 and above liver injury (34). Additionally, a study by Takeshi Hatanaka et al. reported that patients with a CRAFITY score of 0 had the lowest incidence of AEs, followed by those with scores of 1 and 2 (11). Similar conclusions were drawn by Lijie Zhang et al. (35).

Cutaneous adverse events are also non-negligible side effects associated with the use of pembrolizumab for cancer treatment. Studies have indicated that patients with lung cancer may develop severe dermatological conditions such as eczema and psoriasis following treatment with pembrolizumab. These symptoms can be alleviated upon discontinuation of the drug and subsequent symptomatic management (36).

In addition, we also analyzed other inflammatory prognosis scores composed of systemic inflammatory response factors, and although these scores showed strong predictive power in cancer prognosis, the CRAFITY score was more powerful in predicting cancer prognosis from the time-dependent ROC curve.

The study has some limitations. First of all, this study is retrospective and the sample size is limited, selection bias still exists. Secondly, this study was not a multicenter study, so there was no external cohort to verify the predictive value of CRAFITY score in HCC patients treated with Len-P. Large-scale prospective multicenter randomized clinical trials are needed to validate our findings.

## Data Availability

The original contributions presented in the study are included in the article/**Supplementary Material**. Further inquiries can be directed to the corresponding authors.

## References

[1] Siegel RL Giaquinto AN Jemal A. Cancer statistics, 2024. CA Cancer J Clin. (2024) 74:12–49. doi: 10.3322/caac.21820 38230766

[2] Rumgay H Arnold M Ferlay J Lesi O Cabasag CJ Vignat J. Global burden of primary liver cancer in 2020 and predictions to 2040. J Hepatol. (2022) 77:1598–606. doi: 10.1016/j.jhep.2022.08.021 PMC9670241 36208844

[3] Wang L Wang F-S. Clinical immunology and immunotherapy for hepatocellular carcinoma: current progress and challenges. Hepatol Int. (2019) 13:521–33. doi: 10.1007/s12072-019-09967-y 31352593

[4] Vogel A Meyer T Sapisochin G Salem R Saborowski A. Hepatocellular carcinoma. Lancet. (2022) 400:1345–62. doi: 10.1016/S0140-6736(22)01200-4 36084663

[5] Al-Salama ZT Syed YY Scott LJ. Lenvatinib: A review in hepatocellular carcinoma. Drugs. (2019) 79:665–74. doi: 10.1007/s40265-019-01116-x 30993651

[6] Zhu AX Finn RS Edeline J Cattan S Ogasawara S Palmer D. Pembrolizumab in patients with advanced hepatocellular carcinoma previously treated with sorafenib (KEYNOTE-224): a non-randomised, open-label phase 2 trial. Lancet Oncol. (2018) 19:940–52. doi: 10.1016/S1470-2045(18)30351-6 29875066

[7] Merle P Kudo M Edeline J Bouattour M Cheng AL Chan SL. Pembrolizumab as second-line therapy for advanced hepatocellular carcinoma: longer term follow-up from the phase 3 KEYNOTE-240 trial. Liver Cancer. (2023) 12:309–20. doi: 10.1159/000529636 PMC10601873 37901200

[8] Finn RS Ikeda M Zhu AX Sung MW Baron AD Kudo M. Phase ib study of lenvatinib plus pembrolizumab in patients with unresectable hepatocellular carcinoma. J Clin Oncol. (2020) 38:2960–70. doi: 10.1200/JCO.20.00808 PMC7479760 32716739

[9] Llovet JM Kudo M Merle P Meyer T Qin S Ikeda M. Lenvatinib plus pembrolizumab versus lenvatinib plus placebo for advanced hepatocellular carcinoma (LEAP-002): a randomised, double-blind, phase 3 trial. Lancet Oncol. (2023) 24:1399–410. doi: 10.1016/S1470-2045(23)00469-2 38039993

[10] Scheiner B Pomej K Kirstein MM Hucke F Finkelmeier F Waidmann O. Prognosis of patients with hepatocellular carcinoma treated with immunotherapy – development and validation of the CRAFITY score. J Hepatol. (2022) 76:353–63. doi: 10.1016/j.jhep.2021.09.035 34648895

[11] Hatanaka T Kakizaki S Hiraoka A Tada T Hirooka M Kariyama K. Prognostic impact of C-reactive protein and alpha-fetoprotein in immunotherapy score in hepatocellular carcinoma patients treated with atezolizumab plus bevacizumab: A multicenter retrospective study. (2022) 16(5):1150–60. doi: 10.21203/rs.3.rs-1318972/v1 35749019

[12] Bruix J Reig M Sherman M. Evidence-based diagnosis, staging, and treatment of patients with hepatocellular carcinoma. Gastroenterology. (2016) 150:835–53. doi: 10.1053/j.gastro.2015.12.041 26795574

[13] Llovet JM Lencioni R. mRECIST for HCC: Performance and novel refinements. J Hepatol. (2020) 72:288–306. doi: 10.1016/j.jhep.2019.09.026 31954493 PMC12452114

[14] Zhao QT Yuan Z Zhang H Zhang XP Wang HE Wang ZK. Prognostic role of platelet to lymphocyte ratio in non-small cell lung cancers: A meta-analysis including 3,720 patients. Int J Cancer. (2016) 139:164–70. doi: 10.1002/ijc.v139.1 26915723

[15] Grenader T Waddell T Peckitt C Oates J Starling N Cunningham D. Prognostic value of neutrophil-to-lymphocyte ratio in advanced oesophago-gastric cancer: exploratory analysis of the REAL-2 trial. Ann Oncol. (2016) 27:687–92. doi: 10.1093/annonc/mdw012 26787231

[16] Okugawa Y Toiyama Y Yamamoto A Shigemori T Ide S Kitajima T. Lymphocyte-C-reactive protein ratio as promising new marker for predicting surgical and oncological outcomes in colorectal cancer. Ann Surg. (2020) 272:342–51. doi: 10.1097/SLA.0000000000003239 32675548

[17] Tan D Fu Y Tong W Li F. Prognostic significance of lymphocyte to monocyte ratio in colorectal cancer: A meta-analysis. Int J Surg. (2018) 55:128–38. doi: 10.1016/j.ijsu.2018.05.030 29807167

[18] Hu B Yang XR Xu Y Sun YF Sun C Guo W. Systemic immune-inflammation index predicts prognosis of patients after curative resection for hepatocellular carcinoma. Clin Cancer Res. (2014) 20:6212–22. doi: 10.1158/1078-0432.CCR-14-0442 25271081

[19] Bruixola G Caballero J Papaccio F Petrillo A Iranzo A Civera M. Prognostic Nutritional Index as an independent prognostic factor in locoregionally advanced squamous cell head and neck cancer. ESMO Open. (2018) 3:e000425. doi: 10.1136/esmoopen-2018-000425 30426973 PMC6212680

[20] McMillan DC. The systemic inflammation-based Glasgow Prognostic Score: A decade of experience in patients with cancer. Cancer Treat Rev. (2013) 39:534–40. doi: 10.1016/j.ctrv.2012.08.003 22995477

[21] Gruber ES Jomrich G Kaider A Gnant M Sahora K Schindl M. The prognostic index independently predicts survival in patients with pancreatic ductal adenocarcinoma undergoing resection. Ann Surg Oncol. (2020) 27:2017–24. doi: 10.1245/s10434-019-08161-6 PMC7210221 31900809

[22] Kitahara H Shoji F Akamine T Kinoshita F Haratake N Takenaka T. Preoperative prognostic nutritional index level is associated with tumour-infiltrating lymphocyte status in patients with surgically resected lung squamous cell carcinoma. Eur J Cardiothorac Surg. (2021) 60:393–401. doi: 10.1093/ejcts/ezab046 33668047

[23] Teng W Lin CC Su CW Lin PT Hsieh YC Chen WT. Combination of crafity score with alpha-fetoprotein response predicts a favorable outcome of atezolizumab plus bevacizumab for unresectable hepatocellular carcinoma. J Hepatol. (2022) 77:S373. doi: 10.1016/S0168-8278(22)01098-4 PMC9077079 35530282

[24] Yang Y Ouyang J Zhou Y Zhou J Zhao H. The CRAFITY score: A promising prognostic predictor for patients with hepatocellular carcinoma treated with tyrosine kinase inhibitor and immunotherapy combinations. J Hepatol. (2022) 77:574–6. doi: 10.1016/j.jhep.2022.03.018 35351522

[25] Llovet JM Castet F Heikenwalder M Maini MK Mazzaferro V Pinato DJ. Immunotherapies for hepatocellular carcinoma. Nat Rev Clin Oncol. (2022) 19:151–72. doi: 10.1038/s41571-021-00573-2 34764464

[26] Akamine T Takada K Toyokawa G Kinoshita F Matsubara T Kozuma Y. Association of preoperative serum CRP with PD-L1 expression in 508 patients with non-small cell lung cancer: A comprehensive analysis of systemic inflammatory markers. Surg Oncol. (2018) 27:88–94. doi: 10.1016/j.suronc.2018.01.002 29549910

[27] Klümper N Saal J Berner F Lichtensteiger C Wyss N Heine A. C reactive protein flare predicts response to checkpoint inhibitor treatment in non-small cell lung cancer. J Immunother Cancer. (2022) 10:e004024. doi: 10.1136/jitc-2021-004024 35292517 PMC8928397

[28] Wang D DuBois RN. Immunosuppression associated with chronic inflammation in the tumor microenvironment. Carcinogenesis. (2015) 36:1085–93. doi: 10.1093/carcin/bgv123 PMC5006153 26354776

[29] Yoshida T Ichikawa J Giuroiu I Laino AS Hao Y Krogsgaard M. C reactive protein impairs adaptive immunity in immune cells of patients with melanoma. J Immunother Cancer. (2020) 8:e000234. doi: 10.1136/jitc-2019-000234 32303612 PMC7204799

[30] Zheng Y Zhu M Li M. Effects of alpha-fetoprotein on the occurrence and progression of hepatocellular carcinoma. J Cancer Res Clin Oncol. (2020) 146:2439–46. doi: 10.1007/s00432-020-03331-6 PMC11804406 32725355

[31] Yang Z Fu Y Wang Q Pan Y Wang J Chen J. Dynamic changes of serum α-fetoprotein predict the prognosis of bevacizumab plus immunotherapy in hepatocellular carcinoma. Int J Surg. (2024). doi: 10.1097/JS9.0000000000001860 PMC11745582 38905506

[32] Atemezem A Mbemba E Marfaing R Vaysse J Pontet M Saffar L. Human α-fetoprotein binds to primary macrophages. Biochem Biophys Res Commun. (2002) 296:507–14. doi: 10.1016/S0006-291X(02)00909-9 12176010

[33] Pardee AD Shi J Butterfield LH. Tumor-derived α-fetoprotein impairs the differentiation and T cell stimulatory activity of human dendritic cells. J Immunol. (2014) 193:5723–32. doi: 10.4049/jimmunol.1400725 PMC4239186 25355916

[34] Guan R Mei J Lin W Deng M Li S Guo R. Is the CRAFITY score a superior predictor of prognosis and adverse events in hepatocellular carcinoma patients treated with locoregional-immunotherapy? Hepatol Int. (2023) 17:1279–88. doi: 10.1007/s12072-023-10535-8 37129721

[35] Zhang L Sun T Sun B Zhang K Zheng Y Li N. Utility and predictive value of the CRAFITY score in advanced hepatocellular carcinoma treated with transarterial chemoembolization plus tyrosine kinase inhibitors and PD-1 inhibitor. BMC Cancer. (2024) 24:223. doi: 10.1186/s12885-024-11936-0 38365678 PMC10870627

[36] Bobeica C Rebegea L Murariu G Dobre M Nechita A Tatu AL. Cutaneous adverse reactions in a lung cancer patient treated with pembrolizumab: A case report. Exp Ther Med. (2021) 23:15. doi: 10.3892/etm.2021.10937 34815767 PMC8593923

