# Supplementary figures and images for: The CRAFITY score emerges as a paramount prognostic indicator in hepatocellular carcinoma patients received Lenvatinib and Pembrolizumab
Source: Front Immunol. 2024 Nov 1;15:1474456. doi: 10.3389/fimmu.2024.1474456 (PMC11563818; doi:10.3389/fimmu.2024.1474456)

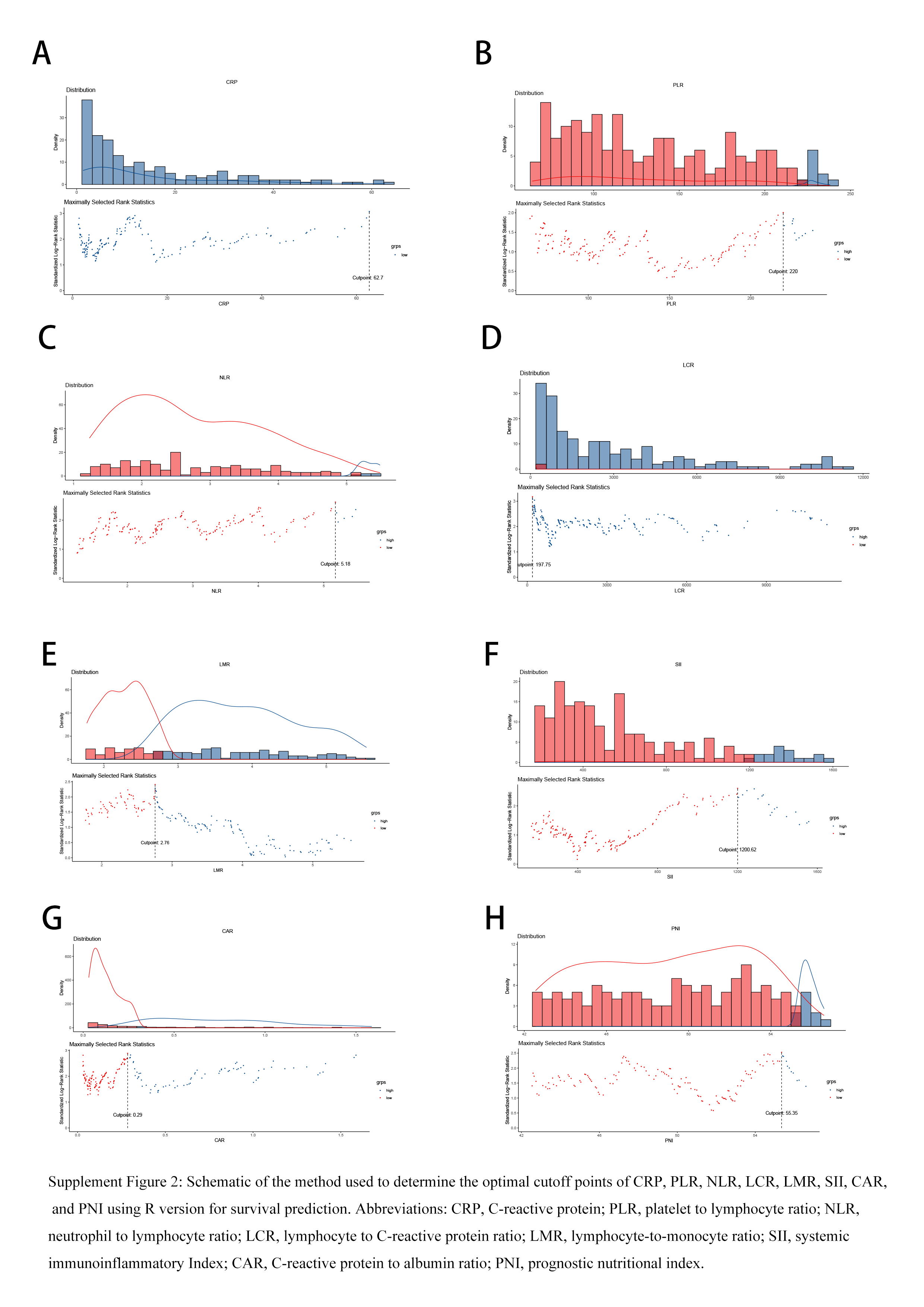

Supplement: Supplementary file 1 [file Image1.tif]

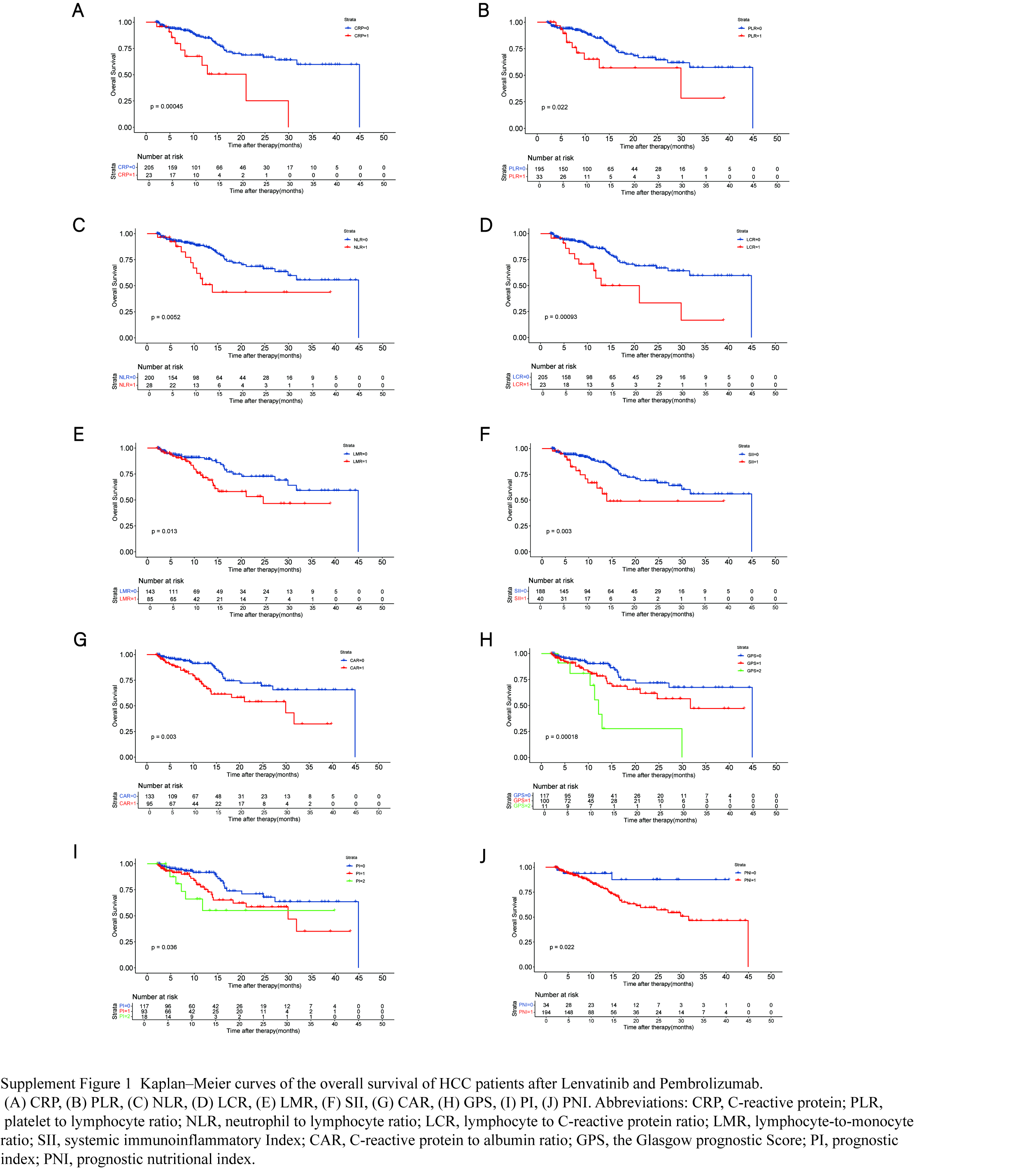

Supplement: Supplementary file 2 [file Image2.tif]
