# Supplementary material for: The CRAFITY score emerges as a paramount prognostic indicator in hepatocellular carcinoma patients received Lenvatinib and Pembrolizumab
Source: Front Immunol. 2024 Nov 1;15:1474456. doi: 10.3389/fimmu.2024.1474456 (PMC11563818; doi:10.3389/fimmu.2024.1474456)
Supplement: Supplementary file 3 [file Table1.docx]

**Table S1** Treatment response of the different CRAFITY score groups.

| **Variables** | **Total** | **CRAFITY 0 points (n=66)** | **CRAFITY 1 points (n=100)** | **CRAFITY 2 points (n=62)** | ***P*-value** |
| --- | --- | --- | --- | --- | --- |
| Overall response | | | | | |
| CR | 6 | 2（3） | 2（2） | 2 (3.2) | 0.868 |
| PR | 57 | 22（33.3） | 30（30） | 15 (24.2) | 0.517 |
| SD | 89 | 17（25.8） | 41（41） | 21 (33.9) | 0.129 |
| PD | 76 | 25（37.9） | 27（27） | 24 (38.7) | 0.199 |
| ORR | 63 | 24（36.4） | 32（32） | 17 (27.4) | 0.556 |
| DCR | 152 | 41（62.1） | 73（73） | 38 (61.3) | 0.199 |
| Intrahepatic response | | | | | |
| CR | 6 | 2（3） | 2（2） | 2（3.2） | 0.868 |
| PR | 63 | 23（34.8） | 33（33） | 17（27.4） | 0.641 |
| SD | 97 | 21（31.8） | 42（42） | 24（38.8） | 0.415 |
| PD | 62 | 20（30.4） | 23（23） | 19（30.6） | 0.453 |
| ORR | 69 | 25（37.9） | 35（35） | 19（30.6） | 0.688 |
| DCR | 166 | 46（70） | 77（77） | 43（69.4） | 0.453 |

**Notes:** Values are presented as the median (range) or n (%). *P*-value < 0.05 is statistically significant.

**Abbreviations:** CR, complete response; PR, partial response; SD, stable disease; PD, progressive disease; ORR, objective response rate; DCR, disease control rate.
