# Supplementary material for: The CRAFITY score emerges as a paramount prognostic indicator in hepatocellular carcinoma patients received Lenvatinib and Pembrolizumab
Source: Front Immunol. 2024 Nov 1;15:1474456. doi: 10.3389/fimmu.2024.1474456 (PMC11563818; doi:10.3389/fimmu.2024.1474456)
Supplement: Supplementary file 4 [file Table2.docx]

**Table S2:** AUROC for the Comparison of the CRAFITY score and Different Inflammatory-Based Scores

| Scores | Progression-free survival | | |
| --- | --- | --- | --- |
|  | 5-Month AUROC | 10-Month AUROC | 15-Month AUROC |
| CRP | 0.530 (47.76-58.20) | 0.581 (53.39-62.81) | 0.581 (54.29-61.95) |
| PLR | 0.509 (45.39-56.33) | 0.628 (57.59-68.05) | 0.585 (54.54-62.44) |
| NLR | 0.516 (46.31-56.81) | 0.613 (56.09-66.53) | 0.624 (57.90-66.92) |
| LCR | 0.527 (47.48-57.92) | 0.567 (51.98-61.50) | 0.592 (55.12-63.36) |
| LMR | 0.506 (43.57-57.55) | 0.600 (54.38-65.53) | 0.668 (61.78-71.78) |
| SII | 0.530 (46.87-59.13) | 0.645 (59.00-59.96) | 0.642 (59.66-68.78) |
| CAR | 0.616 (54.60-68.66) | 0.612 (55.63-66.79) | 0.670 (61.92-72.04) |
| GPS | 0.579 (50.57-65.15) | 0.575 (51.73-63.21) | 0.676 (61.24-73.86) |
| PI | 0.593 (51.55-67.01) | 0.630 (57.07-68.97) | 0.680 (62.93-73.09) |
| PNI | 0.504 (34.36-66.36) | 0.569 (53.79-59.95) | 0.558 (52.27-59.25) |
| CRAFITY | 0.633 (56.85-69.77) | 0.668 (61.32-72.18) | 0.689 (63.85-73.89) |

**Note:** Values are presented as the AUROC (95% confidence interval).

**Abbreviations:** AUROC, area under the receiver operating characteristic curve; CRP, C-reactive protein; PLR, platelet-to-lymphocyte ratio; NLR, neutrophil-to-lymphocyte ratio; LCR, lymphocyte-to-C-reactive protein ratio; LMR, lymphocyte-to-monocyte ratio; SII, systemic immune-inflammation index; CAR, C-reactive protein-to-albumin ratio; GPS, Glasgow prognostic score; PI, prognostic index; PNI, prognostic nutritional index; CRAFITY, Creactive protein and alphafetoprotein in immunotherapy.
